# ECG-Based Biometric Recognition: A Survey of Methods and Databases

**DOI:** 10.3390/s25061864

**Published:** 2025-03-17

**Authors:** David Meltzer, David Luengo

**Affiliations:** 1 Department of Telematics & Electronics, Universidad Politécnica de Madrid, Calle Nikola Tesla s/n, 28031 Madrid, Spain; 2 Department of Audiovisual & Communications Engineering, Universidad Politécnica de Madrid, Calle Nikola Tesla s/n, 28031 Madrid, Spain; david.luengo@upm.es

**Keywords:** ECG biometrics, identification, verification, ECG databases, survey

## Abstract

This work presents a comprehensive and chronologically ordered survey of existing studies and data sources on Electrocardiogram (ECG) based biometric recognition systems. This survey is organized in terms of the two main goals pursued in it: first, a description of the main ECG features and recognition techniques used in the existing literature, including a comprehensive compilation of references; second, a survey of the ECG databases available and used by the referenced studies. The most relevant characteristics of the databases are identified, and a comprehensive compilation of databases is given. To date, no other work has presented such a complete overview of both studies and data sources for ECG-based biometric recognition. Readers interested in the subject can obtain an understanding of the state of the art, easily identifying specific key papers by using different criteria, and become aware of the databases where they can test their novel algorithms.

## 1. Introduction

Biometric traits, both physical and behavioral, are widely used for the recognition of human identity [1]. Several common types of traits are currently used for this, and the benefits and drawbacks of their adoption are well known to date, as stated by Maltoni et al. [2]. Among the biometric traits proposed for more than two decades, the Electrocardiogram (ECG) has been subject to validation as a suitable trait for human recognition since the commonly agreed seminal work by Biel et al. [3].

The ECG is a signal that enables us to observe the electrical activity of the heart through electrodes placed inside the body or (more commonly) on its surface. The heart comprises excitable cardiac cells that produce Action Potentials (AP) in muscle cells to force them to contract. These APs produce voltage variations on the electrodes that can be recorded [4]. The time-based representation of the magnitude of the recorded voltage variations results in a series of waves that include shapes and timings that enable their traditional use in disease diagnosis. In medical diagnosis, it is well known that specific pathologies often show their presence on electrocardiograms [5].

Taking into account the specific characteristics of a biometric trait identified by Maltoni et al. [2], the high *universality* of ECG traits is commonly accepted. As physiological traits, all living beings show them. It is also commonly accepted that physiological traits are less prone to *circumvention* than behavioral traits [6]. Moreover, ECG can be recorded for long periods of time without requiring subjects to show explicit behavior. This is a positive factor for the *collectability* and *acceptability* of this trait. On the negative side, the common use of pairs of electrodes (also referred to as *leads*) to record ECG generally represents a negative factor in terms of the last two characteristics referenced. Another relevant factor that potentially reduces acceptability is related to the fact that ECG can potentially reveal pathologies, even in an environment specifically designed for recognition.

The research studies performed on ECG-based biometrics focus mainly on *distinctiveness*. In general terms, the quantification of a physiological trait must represent the uniqueness of each physical subject. The results of most works to date clearly show that this premise holds, but they do not guarantee perfect identification/verification metrics, revealing that further research needs to be performed.

Another characteristic that represents a source of uncertainty for ECG-based biometrics is *permanence*. It is well known that some physiological traits have high permanence in time as fingerprints. Unfortunately, this does not hold for all physiological traits, and ECG is one of them [7,8,9,10]. For this, there exist mechanisms that can mitigate the negative impact of low permanence for any biometric trait, such as re-enrollment policies.

In this survey, we focus on ECG-based biometric recognition. Two different types of problems are generally taken into account when referring to this term: **biometric identification** and **biometric verification**. According to Wayman [11], the significant amount of development in biometrics in recent decades has created some confusion about the appropriate meaning of both terms. In fact, their use shows a growing amount of ambiguity in written documents such that clarification is clearly needed. At the time of writing, the recommended meanings for “biometric identification” and “biometric verification” according to ISO/IEC 2382-37: 2022 “Information technology —Vocabulary —Part 37: Biometrics” [12] are the following:

**Biometric identification**: The process of searching against a biometric enrollment database to find and return the biometric reference identifier(s) attributable to a single individual.
**Biometric verification**: The process of confirming a biometric claim through comparison.

Although the presented meaning for the term identification seems to be commonly accepted, many contemporary works use different terms with the meaning presented above for biometric verification. *Authentication* has a widespread use as a synonym in a certain number of works subject to this survey, although the aforementioned recommendation does not encourage its use as an alternative to verification.

In more than two decades since the first agreed-upon article on ECG-based biometric recognition, many different techniques have been used to engage the subject. Early works commonly consider the subject as solvable by using probabilistic classifier techniques and interpret it as a Nearest Neighbor classification problem or, alternatively, use well-known Machine Learning (ML) classification algorithms. When using the referenced approaches, feature extraction is commonly treated as an important subject, as well-selected features are known to have a positive impact on recognition metrics. At this point, most studies use fiducial features, as they are commonly used in clinical diagnosis. This requires the delineation of ECG, for which several techniques already exist at this time. Specifically, treating the problem of feature selection and/or reduction has been common since early work. It is not until the problem starts to be approached by using learned feature-based methods that feature selection and/or reduction stops being specifically addressed. With time, non-fiducial features have started to be commonly used in papers. Since then, most works that have used explicit features have used non-fiducial ones. In recent years, it has become more common to find papers using different NNs to solve the recognition problem. In these papers, features are both engineered and learned, depending on the case.

### 1.1. Prior Work

There exist several survey papers that address different aspects of ECG-based biometric recognition to date. The first survey that specifically focused on the subject is the work by Odinaka et al. [13]. Although it was a comprehensive paper that mentioned most works up to the publication date, it is clearly outdated now. This work also presented a fundamental problem with respect to conducting a comparative study of recognition techniques, which is the need for available common ECG databases. In fact, this particular work compared different techniques on a single, private ECG database. Fratini et al. [14] surveyed most works related to ECG-based biometric identification and identity verification (commonly referenced as authentication, especially in the older literature). The work proposed a unified framework to compare different techniques for the first time. It explicitly identified the impossibility of comparing performance metrics when the works used private ECG databases and identified the most widely available publicly available databases at the time. That work is almost ten years old at the time of writing. Pinto et al. [15] published a survey specifically focused on ECG-based biometrics. In their work, a comprehensive list of articles was referenced together with a short list of 12 publicly available ECG databases, some of which were available on PhysioNet [16]. The survey by Rathore et al. [17] was also directly related to our subject, but with a specific focus on different sensing modalities to quantify cardiac activity. Its taxonomy for the cardiac sensing domain broadened the spectrum of sensing modalities beyond the registration of electrical activity. Although the subject was not treated in depth, we should also mention the work by Dargan and Kumar [18]. It surveyed different types of biometric traits, and for ECG-based biometrics, it presented four articles as representative of the main findings in the area up to the date of writing. Finally, it is also worth mentioning the work by Uwaechia and Ramli [19]. It presented the main findings in terms of ECG classification for biometrics, showing the increasing number of works that used NNs for feature extraction and/or classification. It also included a short selection of commonly used ECG databases. Table 1 summarizes previous works that surveyed biometrics using ECG.

Although they referenced some ECG databases, the ECG-based biometric recognition surveys mentioned above did not systematically elaborate a list of the available databases. Since these databases are a fundamental resource for the development of verifiable studies on the subject, Merone et al. [6] reviewed the ECG databases mentioned in works focused on different subjects at the time. They found that only 5 out of the 15 identified databases mentioned in those papers were public. In addition, only two of the five publicly available databases were specifically conceived for biometric recognition. Furthermore, the survey by Flores et al. [20] identified up to eight freely available ECG databases. However, although of interest to our subject, the focus of the work was on cardiovascular diseases. Table 2 shows the aforementioned works that survey ECG databases.

Finally, even though they are only marginally related to the main subject of ECG-based biometric recognition, it is worth mentioning some surveys on ECG analysis which address valuable techniques. The survey by Berkaya et al. [22] presented the works in terms of ECG analysis techniques. Although biometric identification was mentioned, it did not focus on ECG-based biometrics. The work enumerated a list of 21 multi-purpose databases that were available on PhysioNet [16], together with references to articles which had used them. The authors of this work consider the survey in [23] also worth mentioning, as it presented ECG analysis in light of the change of interest that seemed to be occurring from traditional signal processing approaches to ML and Deep Learning (DL) techniques. It is also worth mentioning, despite being centered on analysis techniques, the survey by Merdjanovska and Rashkovska [21]. It also focused on the importance of available ECG databases and included a list of 45 publicly available databases for clinical applications. Finally, the work by Pereira et al. [24] specifically surveyed ECG data acquisition methods, given their impact on biometric recognition. Table 3 shows the works mentioned that survey ECG analysis and acquisition.

### 1.2. Paper Contributions

This work offers a detailed survey of existing studies on ECG-based biometric recognition systems for identification and identity verification. It also offers a detailed survey on the ECG databases available and mentioned in the referenced studies. To the best of our knowledge, a comprehensive survey that covered both the existing articles and the databases used was still missing. This work intends to fill that gap, so that readers interested in the subject can obtain a global understanding of the state of the art and easily find specific papers by using different criteria. This survey contributes to the available literature on ECG-based recognition as follows:

- **Comprehensive and chronologically ordered survey of the literature.**
  In order to fill the existing knowledge gap in ECG-based biometric research, we present a comprehensive and chronologically ordered survey of articles in the area. The main reason for this is the lack of recent works that do so, leaving a time gap for which no survey exists. Other motivations include the importance of identifying each available paper together with the ECG databases that were used in them. In order to provide a deep understanding of the relevance of the obtained metrics in each one, the number of subjects used is shown (when available), together with the referenced metrics. To enable a quick understanding of the techniques used in each paper for feature extraction, reduction, and classification, this information is also provided, if available. In order to facilitate access to all this information, Table A1 provides a thorough compilation of the referenced works, in reverse chronological order from 2001 to 2024. To our knowledge, this is the most complete compilation of ECG-based biometric recognition works available in the literature.
- **Thorough survey of ECGs databases referenced in the literature.**
  We present a thorough survey of ECG databases referenced in the literature. Some researchers have clearly highlighted this necessity given that prior works have only released partial database lists and that only three surveys cover the topic, the latest having been published in 2022. Table A2 provides a comprehensive list of databases used for ECG-based biometric recognition, again in reverse chronological order according to the publication year (or reference to the first mention). In addition to the database name and publication year, other relevant information, such as sampling frequency, number of available leads, length of recordings, and number of subjects, is also included when available.

### 1.3. Paper Organization

This survey is organized in terms of the two main goals pursued in it: first, a description of the main ECG recognition techniques used in the existing literature is performed, including the most relevant references; then, a comprehensive survey of the ECG databases available and used in the aforementioned works is performed.

Section 2.1 describes the different techniques used for feature extraction. Section 2.2 presents the classification techniques used in the literature. Section 3 describes the information that characterizes the databases for their survey. Finally, the tables containing the exhaustive compilation of existing papers and ECG databases can be found in Appendix A and Appendix B, respectively.

## 2. ECG-Based Biometric Recognition Studies

The time period covered by this survey starts in 2001, the year of publication of the seminal article by Biel et al. [3], up to the first semester of 2024. Table A1 contains an extensive compilation of ECG-based biometric recognition works during this period. The works are listed in reverse chronological order, and apart from the year of publication, other relevant data are summarized for each one. The databases used are identified for each work to help the reader understand if replicability and/or result comparison is possible. Moreover, to provide a factor commonly known to have an impact on the recognition metrics, as the different studies did not use the available records of all individuals in many cases, the number of individuals used is provided (if mentioned). The metrics obtained for identification and/or verification are also provided, so that the obtained performance can be compared.

A large number of studies, especially early ones, explicitly use fiducial features, while newer ones commonly use engineered non-fiducial features or even learn them. The authors consider this a relevant decision for each article, and the information provided in Table A1 also includes whether fiducial or non-fiducial features are used, as well as the feature reduction technique, if applied. Another relevant decision in each published study is the selection of the classifier used, which is also provided in Table A1.

Feature extraction and classification techniques have been identified in previous works as two of the most relevant factors to categorize studies. For this reason, the following sections treat them in detail and provide taxonomies for each one.

### 2.1. Extraction of ECG Features

**Figure 1:**
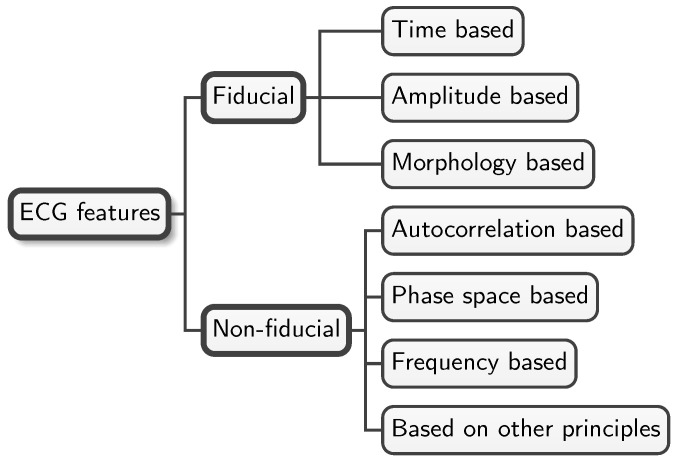
ECG feature taxonomy. This figure shows a simple taxonomy of commonly used features in the biometric recognition literature. Fiducial features shown in the upper part of the taxonomy include three basic types identified by the nature of the points considered: time-, amplitude-, or morphology-based features. The lower branch of the taxonomy includes all the methods used to extract non-fiducial features identified in the literature.

There are a large number of features that can be extracted from ECGs. The literature accepts the separation of these features into two main groups: *fiducial* and *non-fiducial*, as shown in Figure 1. **Fiducial features**, also called *handcrafted or engineered features*, are extracted from the ECG by direct observation and represent specific identifiable elements of the heartbeat that are based on time, amplitude, or morphology. Time-based features refer to the time distance between pairs of well-known ECG points: peaks and onset/offset points of the P and T waves, and the QRS complex. **Non-fiducial features**, on the other hand, include all those features that are not identified as fiducial. Using alternative features overcomes the main problem of fiducial ones, which is a certain amount of uncertainty emerging from the algorithms used [25]. A third group that is sometimes identified in the literature is called **hybrid** (or partially fiducial) features. Hybrid methods combine the non-fiducial extraction of features with the need to identify at least one fiducial point (typically the R peaks).

*ECG delineation*, also known in the early literature as *ECG segmentation* (no to be confused with the segmentation stage in ECG processing), is the processing of the ECG to extract *fiducial features* by direct observation of three main types of elements: time, amplitude, and morphology. The upper branch of Figure 1 shows these three types of fiducial features, while the lower one shows non-fiducial features. Obtaining specific features by ECG delineation is useful not only for biometrics but also for other applications, such as diagnosis in clinical settings.

Table 4 provides a nonexhaustive list of the advantages and disadvantages of using fiducial and non-fiducial features in ECG-based biometric recognition.

#### 2.1.1. Fiducial Features

**Figure 2:**
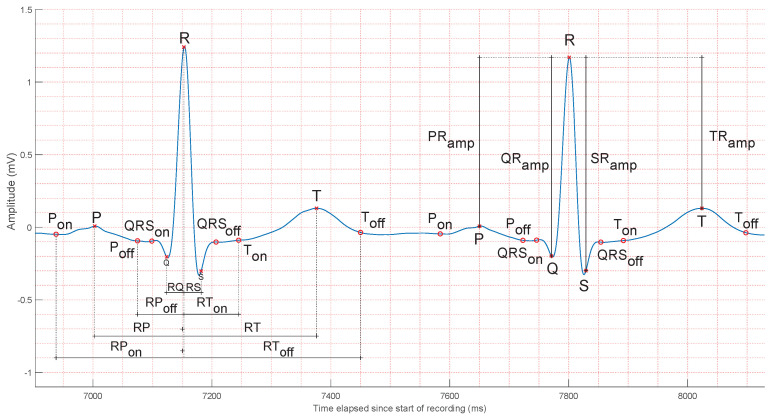
Common ECG fiducial features. The left heartbeat shows time-based features, while the right one shows common annotated amplitude features. Measuring time or amplitude by taking as reference the R peak, as shown, is common in the literature, but it is not the only method used. Alternatives include, for example, measuring time between the onset and offset of specific waves. The ECG segment in the figure is taken from the *V*4 lead of an actual patient: patient #31, record s0100lre, of the PTB database [33].

The left heartbeat in Figure 2 shows eight common time-based features used in different studies. P and T waves, QRS complexes, PR and ST segments, and the widths of the PT and QT intervals have been typically used. Another feature that has been frequently used for biometric recognition is the R-R interval. Some of the aforementioned intervals, specifically the R-R interval and the durations of P and T waves, have an important amount of variability when the heart rate changes. For this reason, some works rely on normalized time-based features to represent a percentage of a heartbeat instead of an interval in seconds [3,28,29,30,31,32].

Several studies state that ECG amplitudes change among individuals [31,34]. Although this fact has been validated, intraclass variance is still a subject of study. The right heartbeat in Figure 2 shows four common amplitude-based features. Their quantification relies on the removal of the existing baseline wander from the ECG, as all the measures compare their mutual positions with respect to the R peak. Other measures of amplitude include the ST segment amplitude [3], the amplitudes of the first and second derivatives of the ECG [35,36], slope and angle features [37], and even the ratios of the described features, representing alternative ways of normalization.

Both time- and amplitude-based features result from a more or less direct observation of the ECG, and their values can be directly inferred from it. A third indirect alternative has been considered to extract features from the ECG or parts of it. It basically consists in obtaining them as a result of processing the ECG, which is sometimes not trivial. A simple method consists in quantifying the slopes between waves and angles in the QRS complex [32]. Another technique is based on the calculation of the average value of certain parts of the ECG, for example, the QRS complex, with respect to multiple other parts of the heartbeat [38,39,40,41,42,43,44]. Table 5 shows a list of fiducial features that have been used in the biometric recognition literature to date [45,46].

As mentioned above, some studies take into account that most fiducial feature values are a function of heart rate. Although this can be easily noticed for time-based features, amplitude- and morphology-based features can also be affected by the heart rate, as it fluctuates with physical activity, strong emotions, or drug consumption. Many **normalization strategies** have been proposed on a per-feature basis [31], full heartbeat resampling [47], partial QT interval normalization [36,45,48,49], R centering by using different resampling ratios on the left and right sides of R [50], and independent wave resampling and recombination [39].

The extraction of fiducial features is a mature subject, where there is a large number of existing studies based on different principles: heuristics using different transforms [51,52,53,54,55,56,57,58,59,60], windowing algorithms [61,62,63], time-domain methods [64,65], mathematical morphology-based approaches [66,67,68], Machine Learning techniques [69,70,71], or algorithms based on Neural Networks [72]. The work by Wasimuddin et al. [23] can serve as a reference for a comprehensive study of this task. However, even if this subject can be considered mature, to date, it is still not fully understood, given the complexity and variability of ECGs, the different environments for ECG extraction, and the absence of a formal scheme for signal acquisition [23,73,74]. A comparison of several techniques for the extraction of fiducial features has been performed in [25], concluding that heuristic methods produce less precise results than wavelet-based and statistical methods.

Heart Rate Variability (HRV), characterized by the pattern of heartbeat occurrence times, is the subject of study for diagnostic purposes, as it can reveal problems in the nervous system [4]. Multiple causes can produce HRV, among which are ectopic beats and noise bursts. This phenomenon can cause variability in the determination of fiducial points. The work by Beraza and Romero [25] revealed a relevant amount of variability in time-based fiducial points when delineating ECGs with different algorithms. This work also revealed that different algorithms led to substantial differences in delineation. To mitigate the effects of HRV, a number of studies have been carried out. An important milestone was the work by Martínez et al. [52], which used wavelets to represent the ECG, resulting in better results than heuristic-based algorithms. Other alternatives include the work by Akhbari et al. [75], who used a multi-hidden Markov model; Lee et al. [27], who applied a curvature-based vertex selection technique; Akhbari et al. [76], whose work was based on a switching Kalman filter; or Bae et al. [77], who used a one-dimensional bilateral filter detector. Another well-known technique to mitigate the effects of HRV consists in clustering heartbeat morphologies [4]. This method helps to take into account the presence of ectopic heartbeats and other variability sources, so that the fiducial characteristics can eventually be extracted separately for each cluster. For a more in-depth analysis of the subject, the authors refer the reader to the surveys on ECG analysis and acquisition in Table 3.

#### 2.1.2. Non-Fiducial Features

Several techniques to obtain non-fiducial features have been devised to date, and all can be identified in terms of the principles on which they are based, as shown in Figure 1.

**Autocorrelation-based features** are extracted from fixed-length windows of *N* samples taken from the discrete time-based ECG signal segments, which are used to obtain their normalized Autocorrelation (AC). Heuristics are usually applied to determine *N*. According to Plataniotis et al. [78] and Lee [79], a time interval in the range of 5 to 10 s is a good choice for biometric recognition. It is a common technique to follow the extraction of AC series with some dimensionality reduction technique. Discrete Cosine Transform (DCT) is typically used for this [80,81,82,83,84,85,86] (this combination is known as the Autocorrelation/Discrete Cosine Transform (AC/DCT) method), since it has been commonly used for signal processing and data compression [87], but Linear Discriminant Analysis (LDA) has also been used [78,82,83,88].

Alternatively, time-based ECGs can be projected into *n*-dimensional spaces by means of a *time-delay technique*. This technique is based on considering the sequences of samples of ECG windows, s[i], and the delayed sequences of samples obtained through time shifting, s[i+Δ]. The first works using this technique for ECG-based biometrics were performed by Fang and Chan [89,90]. In these works, time delay was used to derive three time-shifted ECG sequences from 5 s windows (also called *epochs*), using delays between 4 ms and 36 ms. Those three sequences were then used to build a normalized three-dimensional trajectory in the **phase space** that could be used as a feature. Similar alternative techniques have also been devised, using three different leads instead of time-shifted ECG sequences. The reduction in features in the phase space is performed by discretizing the state space by using a cubic grid M×M×M and annotating the cells that are crossed by the trajectories to construct a *coarse-grained structure*. Recognition is then based on structure comparison methods [40,91,92].

Another well-known alternative to search for non-fiducial ECG features is to elaborate on **frequency-based features**. Several studies have been performed in this area. Coefficients of the Fourier transform were used as features by Saechia et al. [93], the Mel-Frequency Cepstrum Coefficients (MFCCs) [94] were used in two studies [95,96], other authors used procedures similar to the Hilbert–Huang transform over the Empirical Mode Decomposition (EMD) of ECG segments (epochs) or a modified version of it to obtain features [97,98], and Loong et al. [99] used the first 40 points of the linear predictive coding (LPC) spectrum as features for overlapped 5 s ECG windows.

Finally, there exist studies **based on other principles** in addition to the basic ones presented in the taxonomy in Figure 1. In some cases, the research work has been directed to extracting features in multi-lead scenarios [89,100], others have used feature extraction techniques such as Cepstral Analysis [101,102], Piecewise Linear Representation (PLR) [103], Pulse Active Ratio [104], autoregressive coefficients and mean of power spectral density (PSD) [105], compressed ECG [106], wavelet coefficients [107], and even combinations of known methods such as wavelet decomposition, frequency analysis, and correlation coefficients [108,109].

#### 2.1.3. Features in Neural Networks

Although NNs and DNNs were seldomly used techniques in early works, more recent studies have incorporated them more frequently. Basic cases included those where a high number of ECG signals were available or for niche applications, such as continuous identification or identity verification [110,111,112,113,114,115,116,117,118,119]. Their use commonly avoids the need to devise specific methods to analyze ECGs and extract features in a process commonly referred to as “feature engineering” [120]. It should be noted that in some cases, these works mention the need for a large number of ECG signals for training purposes [121] and even the need to extend the ECG datasets by data augmentation [74,120,122,123], using DGMs [124,125] or even auxiliary classifier GANs [126].

As NNs for ECG-based biometric recognition are used more often, a new type of classification has emerged to identify methods that lead to the acquisition of characteristics: *manual feature-based methods and methods based on learned features* [127]. **Manual feature-based methods** are those that generate both fiducial and non-fiducial features, as previously presented in this section. They assume that all ECGs share common characteristics that can be reflected in distinctive features. Unfortunately, this assumption does not always hold for all ECGs. For example, not all heartbeats of a subject can be considered similar, especially when there is a pathology, such as an ectopic heartbeat arrhythmia [128]. It can be verified by inspecting Table A1 that most of the early biometric recognition studies use sets of individuals who do not suffer from heart pathologies or use selected individuals with minor arrhythmias given the lack of other alternatives. When pathologically affected individuals are used, performance tends to decrease [104,107,113]. **Learned feature-based methods** are conceived to overcome the proven fact that not all ECGs share common characteristics [127,129]. They try to learn the structural and hierarchical characteristics of actual ECGs in order to extract representative features by generalization [127]. Although learned feature-based methods have NNs, and specially CNNs, as a natural environment for development, other ML techniques have also been used [112,130,131,132,133,134,135,136,137]. It should be mentioned that until very recently, there were no available studies using autoencoders for feature extraction [117,121,138,139,140].

#### 2.1.4. Learned Features and Fiducial Points

It is particularly interesting to draw attention to the fact that most studies that use non-fiducial or learned features still rely on a segmentation stage that requires at least a minimum number of fiducial points [32,39,40,48,85,89,102,107,108,111,112,115,129,136,141,142,143,144,145,146,147,148,149,150,151,152,153,154,155,156,157,158]. This underscores the continued significance of a delineation stage that, even if it is not used for the direct generation of fiducial features, serves as assistance for a further feature extraction stage. As an example, in one of the first works in this sense, Irvine et al. [129] used Principal Components Analysis (PCA) for feature extraction from the samples of each heartbeat. To achieve this, segmenting the ECG signal into heartbeats by identifying a certain number of fiducial points through delineation was previously needed.

### 2.2. Classification Techniques

**Figure 3:**
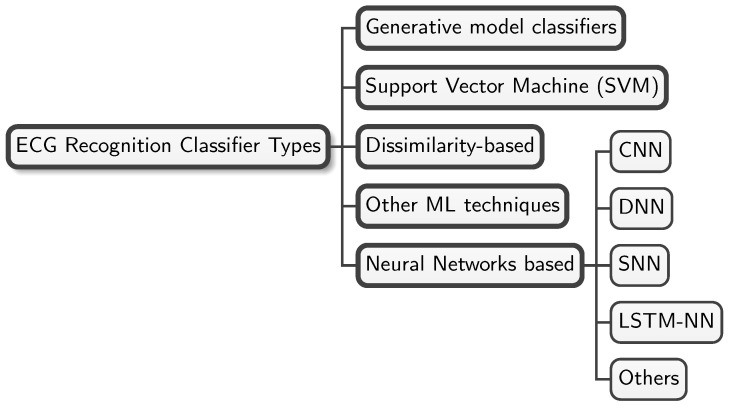
Classifier taxonomy. This figure shows a concise taxonomy of the most commonly used classifier types in the ECG-based biometric recognition literature.

There are a large number of classifier types used to date for ECG-based biometric recognition in the literature. Figure 3 shows a concise taxonomy of the ones that are most commonly used in the area.

**GMCs** learn the characteristics of each class by using training data, thus enabling the determination of potential feasible input data corresponding to each one. The seminal work by Biel et al. [3] used the Soft Independent Modelling of Class Analogy (SIMCA) classifier. It modeled each class independently and belonged to this category. The works by [28,31,35,44,47,148,153,159,160,161,162,163] used LDACs, which are also GMCs. Additional studies using GMCs include [161], which used the naive Bayes classifier, and [78,108,161], which used the Log-Likelihood Ratio (LLR).

**Support Vector Machine (SVM)** is another commonly used type of classifier for ECG-based biometrics. It projects feature vectors into a high-dimensional space in order to find the hyperplanes that represent the boundaries between classes. Nonlinear classification is possible through kernel functions. To the best of the authors’ knowledge, the first work using the SVM classifier in ECG-based biometrics was the one by Li and Narayanan [102]. Since then, many other works that use SVMs have been published [44,107,152,156,157,164,165,166,167,168,169,170,171,172,173,174].

**Dissimilarity-based classifiers** compute dissimilarity metrics between a feature vector that must be recognized and a set of labeled vectors obtained during the training phase. A subset of *k* labeled vectors is determined on the basis of the minimum dissimilarity with the vector to be recognized, and finally, a single (or multiple) label is predicted. *k*-NN is the most common ML algorithm that behaves as described. This type of classifier has been commonly used in a large number of works on the subject since 2001 to date, as can be verified by evaluating Table A1: [32,38,39,40,45,82,83,85,89,104,105,113,117,129,136,139,143,150,151,153,156,157,163,164,170,175,176,177,178,179,180,181,182,183,184,185,186,187,188]. Many of the referenced works implemented the simplest form of the *k*-NN classifier, where k=1. Indeed, they rarely mention *k*-NN, denoting it as the *nearest-neighbor criterion classifier* instead. One of the works that explicitly mentioned using *k*-NN with k>1 was the one by Gürkan et al. [176]. It is worth mentioning that not all works that used the *k*-NN classifier were conceived to use the complete training set of feature vectors to obtain predictions. Some of the works used a subset of *prototype features*, derived from the training set, thus achieving different advantages [104,136,142].

Using **Neural Network (NN) classifiers and/or feature extractors** represents a relevant trend in this area. Several works have used NNs for ECG-based biometrics since the early days of ECG-based biometric recognition. In the period between 2002 and 2019, we have found 16 works that used them [30,48,93,99,105,111,112,115,138,145,147,155,157,166,189,190]. By inspecting Table A1, it can be verified that the number of works that use NNs has recently increased significantly. As an example, since 2020, 30 out of the 44 works identified on ECG-based biometrics have used NNs, either alone or together with other types of classifiers and/or feature extractors [118,121,122,125,127,158,171,173,174,182,191,192,193,194,195,196,197,198,199,200,201,202,203,204,205,206,207,208,209].

The works in the literature use different types of NNs. Figure 3 shows the most commonly used ones: CNNs, DNNs, RNNs, SNNs, and LSTM-NNs.

**CNNs** are, by far, the most commonly used NNs in this area [112,115,118,121,122,127,158,173,191,193,195,196,200,202,203,204,205,206,207,209]. In some works, features are engineered to accommodate 2D matrices, so that 2D CNNs can be used as classifiers. Occasionally, spectrogram images of ECGs have been used, as in the works by da Silva Luz et al. [112], Ammour et al. [203], Aleidan et al. [210]. In other cases, plain 2D images of ECGs were used [194,204,205], as well as Gramian angular fields [206] or other engineered 2D features [155,192,193].

**DNNs** have recently been incorporated for ECG-based biometrics, having a limited number of references at the time [125,174,191,194].

Another interesting type of NN are **SNNs**. They have also been used in some works, as in Ibtehaz et al. [158], Tirado-Martin and Sanchez-Reillo [192], Prakash et al. [204].

Since an ECG is a time-based signal, several works have used **LSTM-NNs** as classifiers for ECG recognition, as in Prakash et al. [118], Tirado-Martin and Sanchez-Reillo [192], Jyotishi and Dandapat [199]. **RNNs** have also been used, as in the works by Salloum and Kuo [111], Kim et al. [122]. Continuing with the consideration of the recognition of a time-based signal, the publication by Aslan and Choi [208] uses **GINs**.

Worth mentioning also are the works by Sepahvand and Abdali-Mohammadi [182], Chee and Ramli [197], which use plain fully connected NNs, attaining good performance metrics. Another interesting line of work uses **PNNs**, as in the publications by Ghofrani and Bostani [105], Li et al. [198]. Finally, it is also worth noting that **Autoencoders** have started to be used in this area, both for automated feature extraction and together with other types of classifiers [117,121,138,139].

In addition to the most common classification techniques previously described, ECG recognition studies have used a larger variety of classifiers. Decision Tree (DT) classifiers were used by Aziz et al. [168], Hwang et al. [170], Bhuva and Kumar [173]. The Random forest (RF) classifier was used in the works by Fatimah et al. [172], Carvalho and Brás [211]. LDA and Quadratic Discriminant Analysis (QDA) classifiers have also been used by Zhang and Wei [161] and Sarkar et al. [153], respectively.

**Figure 4:**
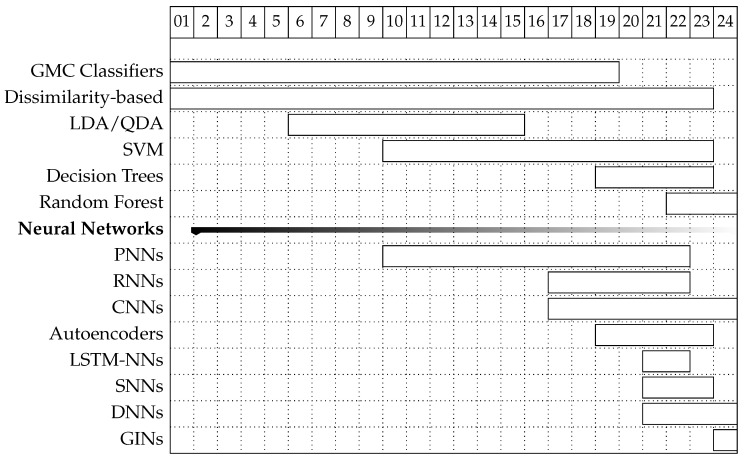
First and last appearance of classification techniques according to the surveyed studies. Earlier techniques used are depicted higher than those which appeared later.

A depiction summarizing the time evolution regarding the use of each classification technique with horizontal bars is shown in Figure 4. The leftmost part of each bar indicates the year of the earliest dated work identified by using each classification technique. The rightmost part of the bar indicates the latest study date available so far. NN-based classification is considered a single group with works dating from 2002. Within this group, the most relevant techniques are also identified below the group bar.

## 3. ECG Databases

Research involving ECGs requires the collection of actual recordings to be carried out, and the most convenient environment to obtain them is the clinical environment, where electrocardiographs are commonly used on a daily basis. Due to their immediate proximity, clinical research can be the primary beneficiary of the acquired ECG data. However, there exist a number of reasons that favor the publication of sets of ECG records, also called **ECG databases** or **ECG datasets**, in the available literature: the replicability of original studies and the comparison of results obtained by using different techniques and common databases are among the most relevant ones.

Table A2 shows a comprehensive compilation of existing ECG databases that have been mentioned in ECG-based biometric recognition studies. As shown in Table 2, only three previous works have aimed to list available ECG databases, and none of them has done so comprehensively [6,20,21]. In fact, the most recent survey presented a list of 45 databases, while our work identifies and lists 74. In order to ease its use, a database should be easily available. In this sense, some of the presented databases are publicly available, others are only available upon request, and finally, some of them were collected for specific studies and are not available to date. Some databases contain exclusively ECGs, while others store ECGs together with other signals. Databases containing different types of signals are usually intended for multivariable or fusion research in emotion recognition or polysomnography studies.

Table A2 has been ordered so that newer databases are shown first. This helps identify the fact that new databases are still being published and mentioned in papers to date. The authors interpret this as reflecting the fact that there is no common agreement on a set of reference databases to use for study comparisons and reproducibility in the area. By inspecting Table A2, the databases most frequently used in the literature are MIT-BIH (one or more of the ten available), PTB, and ECG-ID. In addition, it can be seen that only 9 out of the 67 databases identified contain records from more than 1000 subjects. This is consistent with the fact that although electrocardiograph devices are widely available today, both in clinical and research environments, collecting a large number of ECGs and compiling them into a database requires considerable effort.

The databases have been considered in terms of the more relevant variables to evaluate the extent of the works that use them, as well as for comparison purposes: the ECG *sampling frequencies*, the *recorded leads*, the *time length* of the recordings, and the *number of subjects* (not to be confused with the number of available records in the database). The Analog-to-Digital (A/D) processing of ECG recordings has made it possible to parameterize the signal capturing fidelity in terms of reduced quantization noise and captured frequencies. Current digitization devices use a minimum of 12 bits for the digital conversion of samples. Taking into consideration that some ECG waves have an amplitude of a few millivolts and that a maximum of a 1-volt dynamic range can be achieved (without taking into consideration the wandering error that can be removed), this sets a maximum on the quantization noise of LSBmaxerror=1212=0.24 µV, enough for the dynamics of the signals under study and to identify low-amplitude waves such as P or T [212]. Indeed, newer databases typically use 12, 15 or 16 bits for quantization.

Regarding the ECG sampling frequency, the established literature considers that while a wide range of sampling rates can be used, 500 samples/s is the minimum rate that should be used [4]. Lower sampling rates can be used, but this factor can decrease performance, as there is a loss of fine details in the recordings [213]. If low-sampling-rate databases are definitely needed, downsampling can be applied as in, for example, the works by Gong et al. [125], Censi et al. [212].

It seems clear that using a higher number of leads (for example, a 12-lead configuration) can improve recognition metrics [213]. However, single-lead configurations have also been shown to be valid for biometric recognition [214]. The works referenced in Table A1 use different configurations with single or multiple leads for recognition. Non-conventional leads, placed on the upper arm, behind the ears, on the wrist, or on the fingers, have also been tested, with promising results [215,216,217]. Unfortunately, using more than one (or two) leads restricts the application of biometric recognition to clinical scenarios, where multi-lead configurations are common. In terms of acceptability, **off-the-person** single-lead configurations are much simpler and are perceived as more acceptable in common scenarios compared with multi-lead configurations, also commonly known as **on-the-person** configurations. For an in-depth taxonomy of ECG data acquisition systems, the authors refer the reader to the work by Plácido da Silva et al. [218]. The current literature favors off-the-person techniques that enable better acceptance by individuals, as well as short setup times and simplicity, typically implementing *continuous verification* (identity verification in wearable/portable devices) [115,156,173,219,220,221,222,223,224].

Short-term ECG data (less than several minutes) are commonly used for clinical diagnostic purposes, as they regard a reasonable time for the detection of most cardiac diseases. On the other hand, long-term ECG data are needed for the diagnostic of diseases that intermittently express themselves in ECGs, such as paroxysmal ventricular fibrillation or atrial fibrillation. Considering biometric recognition, it is commonly considered that shorter ECG segments lead to lower recognition metrics. In this sense, Ramos et al. [225] recently mentioned the lower limit of 10 s as the minimum length of the ECG segment that contains enough information for biometric recognition. Determining optimal ECG segment size is still a pending task, as it appears to depend on several factors, such as the quality of the acquired data, preprocessing, feature extraction, and classification techniques. While most available databases to date collect short-term ECG data and long-term ECG data for different diagnostic purposes, some of the newest databases offer ECG segments between 10 and 20 s long, such as PTB-XL, the Lobachevsky University Electrocardiography Database (LUDB), the Chapman University and Shaoxing People’s Hospital database, Motion Artifact Contaminated ECG, and the ECG-ID database. At the time of writing, it can be concluded that the lengths of the recorded segments in most available databases convey enough information for biometric recognition purposes.

One of the most relevant parameters for deciding which database to use for biometric purposes is the number of different subjects whose ECGs have been recorded. The literature on ECG-based biometric recognition uses readily available databases whose conception was mainly for clinical research. Those databases usually contain records of a number of different subjects that typically are in the tens or a few hundreds at most, as shown in Table A1. An intuitive approach to biometric recognition states that recognition performance metrics should decrease as the number of different subjects increases. This fact has been validated by experiments with readily available databases in the work by Jekova et al. [214]. Another important criterion to consider when selecting a database is its use in previous research. Choosing a database that has been previously used for biometric recognition experiments can be positive when comparisons of results are of interest.

## 4. Discussion

There exist many published studies that involve identification, verification, or both problems at the same time. Most of these studies claim to achieve excellent performance metrics. For identification purposes, the most common performance metric used is *Accuracy (ACC)*. Early studies sometimes express identification performance in terms of other ratios, and newer studies sometimes mention metrics that can quantify the effect of training imbalance, such as *precision*, *recall*, or *F-score*. On the other hand, all studies that treat identity verification use the *Equal Error Rate (EER)* metric.

The authors consider it to be extremely relevant that the vast majority of studies use sets with only tens or a few hundreds of individuals in their experiments. Early works typically used a few tens of individuals, while more recent works show a clear trend to include numbers in the hundreds. Only a limited number of studies, especially newer ones, report metrics resulting from experimenting with numbers of individuals in the thousands. As the number of individuals increases, studies can evaluate how well their techniques behave in the presence of factors that such as class separation or intra- and interclass variability. A contributing factor to the small number of individuals in most studies is the restricted access to extensive databases with records for many individuals. With a few exceptions, such as the PTB-XL database, there were no publicly available databases with records from thousands of individuals until recently. Another relevant factor preventing studies from trying to experiment with large numbers of individuals is the intention of the recorded ECGs. To date, most databases with records from a high number of individuals exist for clinical purposes. Their records usually show pathologies, with the ECG signal sometimes behaving far from a Normal Sinus Rhythm (NSR) and eventually resulting in a negative impact on recognition metrics. In this sense, ECG-based biometric recognition techniques should take into account that aside from specific cardiac affections that require clinical intervention, many healthy subjects also present sporadic irregularities (such as ectopic beats) that should be acceptable for recognition techniques. It is also important to note that in a large number of studies, the ECG sets are selected from healthy individuals when possible or from individuals who show specific homogeneous pathologies, such as arrhythmias. From the study of the available papers and databases used, the authors consider that further work is needed to determine the effects of existing pathologies on recognition metrics.

With the increasing number of studies that use NN-based techniques, the composition of ECG databases, both in terms of number of individuals and length of records, becomes increasingly important. On the one hand, it can be shown from the metrics in Table A1 that NN-based classifiers can obtain excellent recognition metrics with hundreds of individuals. Unfortunately, there are only a few studies that use sets with thousands of individuals. On the other hand, the accepted principle that short records are sufficient for experimentation on ECG-based biometrics poses a challenge for training NN-based classifiers. This has resulted in a recent body of literature that uses data augmentation, DGMs, or GANs for coping with short-time recordings of each individual in databases and the need for enough examples to train the models.

To summarize the problem posed by the availability of databases, the work by Barros et al. [226] is an example of a study that recognizes this fact: “*Existing works on ECG for user authentication do not consider a population size close to a real application*”.

Assessing the characteristics of the databases used by studies over time, it is feasible to have an approximate idea of future needs and requirements and anticipate potential trends in future databases. The motivation to validate novel recognition methods with an increasing number of individuals closer to real applications will likely result in new databases with recordings of individuals in the thousands and even tens of thousands. The same motivation will probably result in the availability of databases with higher ratios of healthy versus pathological subjects to try to meet the expected ratios of real applications. This may even result in more initiatives to compile datasets in non-clinical environments. Another factor that may impact the configuration of future databases is the increasing number of studies that use NNs for biometric recognition, as shown in Figure 4. This will require an increasing number of individuals and longer records available for each one, to avoid the need for augmentation techniques to obtain an acceptable number of training data. Finally, with the emergence of new applications that rely on signals obtained from one or two leads at most, the authors believe that an increasing number of the new databases will include recordings obtained with configurations other than those used for medical purposes.

One of the most evident applications of biometric recognition is recognition in clinical environments (also known as *patient identification*), where acceptability does not represent an issue, as ECG monitoring is commonly used [227]. Consequently, an interesting application domain that has a high potential for development is continuous identity verification (also known as *continuous authentication*). This type of application seems to be gaining relevance, encompassed with ambient intelligence and wearables [110,111,112,113,114,115,116,117,118,119,173]. A tangible example of a continuous identity verification scenario was presented in the work by Lourenço et al. [215], where ECGs were recorded by electrodes placed on the surface of a steering wheel. Another scenario was shown by Kim et al. [217]. In this study, multimodal biometrics were used, capturing the photomatrix and ECGs by means of a wrist band.

Another relevant fact arising from the available literature is that most of the works have a batch-based approach to the problem. The work by Wang et al. [183] is the only one to apply Online ML techniques. Given the state of the art, as well as the available databases, the authors consider that the validation of the ECG as a biometric trait for a realistic number of individuals is still a pending issue. A further challenge for practical applications will be the need to maintain ECG-based biometric systems with a very large number of enrolled individuals and the need to enroll and disenroll them. This represents an important issue if batch-based techniques are used, possibly forcing researchers to use alternative approaches, such as online techniques.

Finally, there are no specific works that focus on the impact on the performance of the variance in ECGs over time (*permanence*). As a physical-based trait, the ECG will change when the physical characteristics that produce it are modified. Some studies try to take this short-time factor into account by using different recordings of the same individuals in training and evaluation. Again, this type of studies cannot be specifically conducted until long-term record databases of the same individuals exist. Moreover, for the purpose of studying the effect of aging, those intervals should last years. For a more in-depth knowledge of the current work about aging in the ECG, the authors refer the reader to the survey by Ansari et al. [228].

The authors have identified two basic scenarios for which the surveyed ECG databases were originally intended. Historically, the most natural scenario has been the *clinical environment*. Common applications here were clinical decision support, research on multiple cardiac conditions (arrythmia, assumption of drugs, etc.), development of automatic ECG analysis methods, and long-term longitudinal studies. Most databases referenced in the studies were originally intended for use in this scenario. The databases most used in studies in this scenario are those freely available: the MIT-BIH group of Physionet databases [16,229,230,231,232,233,234,235], the PTB Diagnostic ECG Database [33], and the QT database [236]. Besides the referenced ones, it is worth mentioning the ECG-ViEW II database [237], not used for ECG biometric recognition to date but relevant for research related to permanence. Within the clinical scenario, patient identification has emerged as a new application, as explained above.

The focus of this survey is the second scenario: *general biometric recognition* using ECGs. Only a few databases originally include biometric recognition among their goals: Check Your Biosignals Here initiative (CYBHi) database [238], University of Toronto ECG Database (UofTDB) [239], ECG-ID database [240], Chosun University CU-ECG [171,208], or Heartprint database [241]. In any case, various databases not originally designed for biometric applications have also been frequently used for applications in this scenario, such as the PTB Diagnostic ECG Database [33], the MIT-BIH Arrhythmia Database [229], or the ECG-ID Database [240].

## 5. Conclusions

ECG-based biometric recognition is still a very active area of research, despite the fact that there are studies in the area dating back more than two decades. To date, there are no other works that fully cover both the studies performed on the subject and the ECG databases used in the area. This survey provides a comprehensive overview of both. It enables readers to obtain an understanding of the state of the art, identify the types of recognition techniques applied over time, search for specific studies by using different criteria, and come to know the available ECG databases to choose among them for the evaluation of novel techniques.

The available studies claim to achieve excellent performance metrics in most cases. Unfortunately, the vast majority of these studies use ECG databases with population sizes much smaller than those expected in real applications, thus limiting the validation of their techniques. This survey shows that one factor causing this problem has been the restricted access to databases with a large number of records from many individuals until recently. Another cause is the fact that most of the available databases containing a large number of individuals exist only for clinical purposes.

Another relevant conclusion arising from this survey is related to the evolution of recognition techniques. Although many different methods have been used for ECG-based recognition, most recent studies show a clear tendency to use NNs and, more specifically, CNNs, DNNs, or GINs. However, a thorough comparison of the performance of the Shallow and DL classification methods used, for different databases and application scenarios, is still lacking in the literature.

Finally, note that there are still several open research challenges in the area. The nature of an actual biometric system, which involves the enrollment and disenrollment of large numbers of individuals, seems to be better suited for online techniques than for batch approaches. Unfortunately, only marginal work has been published to date in this area. Another important area of research is the study of the impact on recognition metrics of the variance in ECGs over time caused by aging and the emergence of pathologies. There are also a limited number of studies on this subject.

## Figures and Tables

**Table 1:** Works surveying biometrics using ECG.

Year	Work Title	Brief Description
2012	ECG biometric recognition: A comparative analysis [13]	The first comprehensive survey on ECG biometrics, highlighting the need for common ECG databases and comparing techniques on a private database.
2015	Individual identification via electrocardiogram analysis [14]	Proposed a unified framework for comparing ECG techniques, addressing issues with private databases and identifying publicly available ones.
2018	Evolution, current challenges, and future possibilities in ECG biometrics [15]	Focused survey on ECG biometrics, listing a comprehensive set of articles and 12 publicly available ECG databases.
2020	A survey on heart biometrics [17]	Broadened the spectrum of cardiac sensing modalities beyond electrical activity registration with a focus on sensing techniques.
2020	A comprehensive survey on the biometric recognition systems based on physiological and behavioral modalities [18]	Surveyed various biometric traits, including ECG-based biometrics, referencing four key articles in the domain.
2021	A comprehensive survey on ECG signals as new biometric modality for human authentication: Recent advances and future challenges [19]	Highlighted the increasing use of neural networks for feature extraction and classification in ECG-based biometrics.

**Table 2:** Works surveying ECG databases.

Year	Work Title	Brief Description
2017	ECG databases for biometric systems: A systematic review [6]	Reviewed ECG databases mentioned in papers, finding that only 5 out of 15 identified databases were public, and only 2 were conceived for biometric recognition.
2018	Readily available ECG databases [20]	Identified up to 8 freely available ECG databases, focusing primarily on cardiovascular disease-related applications.
2022	Comprehensive survey of computational ECG analysis: Databases, methods and applications [21]	Focused on the importance of ECG databases, including a list of 45 publicly available databases for clinical applications.

**Table 3:** Documents related to ECG analysis and acquisition methods.

Year	Work Title	Brief Description
2018	A survey on ECG analysis [22]	Surveyed ECG analysis techniques, listing 21 multi-purpose databases available on PhysioNet and referencing articles that use them.
2020	Stages-based ECG signal analysis from traditional signal processing to Machine Learning approaches: a survey [23]	Presented ECG analysis in light of the shift from traditional signal processing to Machine Learning and Deep Learning techniques.
2022	Comprehensive survey of computational ECG analysis: Databases, methods and applications [21]	Focused on the importance of ECG databases, including a list of 45 publicly available databases for clinical applications.
2023	Biometric recognition: A systematic review on electrocardiogram data acquisition methods [24]	Surveyed ECG data acquisition methods and their impact on biometric recognition.

**Table 4:** Advantages and disadvantages of fiducial and non-fiducial features for ECG-based biometric recognition.

Feature Type	Advantages	Disadvantages
Fiducial	Well-defined reference elements. Some features are also used for clinical purposes. Some features can be obtained from medical specialists’ annotations. Can benefit from algorithms that compensate for Heart Rate Variability [26]. Interpretability.	Universality is assumed. Only a limited number of them can be figured out (see Table 5). Accurate localization of fiducial points is still a challenge [25,27]. Susceptible to electrode motion and electromyographic artifacts [4].
Non-fiducial	In some cases, they do not require the detection of ECG waveform boundaries. They reduce error rate and computational effort compared with fiducial features [14]. Do not assume universality in most cases.	Some techniques (semi-fiducial) need at least a single fiducial point to work. Some extraction techniques produce a large number of features that may need reduction. The techniques that use NNs usually need large numbers of ECGs to properly operate. Lack of interpretability.

**Table 5:** List of fiducial features used in ECG biometric recognition works to date.

Feature Group	Description	Identification
Time	Obtained as time differences and ratios between time fiducial points.	$RP_{on},RP,RP_{off},RS,RT_{on},RT,RT_{off},RR,$ $\frac{PT}{QS},\frac{QT}{QS},\frac{RP_{on}}{P_{on}T_{off}},\frac{RP}{P_{on}T_{off}},\frac{RP_{off}}{P_{on}T_{off}},$ $\frac{RS}{P_{on}T_{off}},\frac{RT_{on}}{P_{on}T_{off}},\frac{RT}{P_{on}T_{off}},\frac{RT_{off}}{P_{on}T_{off}}$
Amplitude	Obtained as amplitude differences between amplitude fiducial points.	$P_{a}Q_{a},Q_{a}R_{a},R_{a}S_{a},S_{a}T_{a},Q_{a}S_{a},P_{a}S_{a},P_{a}T_{a},$ $Q_{a}T_{a},\frac{S_{a}T_{a}}{Q_{a}S_{a}},\frac{R_{a}S_{a}}{Q_{a}R_{a}},\frac{P_{a}Q_{a}}{Q_{a}S_{a}},\frac{P_{a}Q_{a}}{Q_{a}T_{a}},\frac{P_{a}Q_{a}}{P_{a}S_{a}},$ $\frac{P_{a}Q_{a}}{Q_{a}R_{a}},\frac{P_{a}Q_{a}}{R_{a}S_{a}},\frac{R_{a}S_{a}}{Q_{a}S_{a}},\frac{R_{a}S_{a}}{Q_{a}T_{a}},\frac{S_{a}T_{a}}{P_{a}Q_{a}},\frac{S_{a}T_{a}}{Q_{a}T_{a}}$
Slope	Obtained on the basis of the slope of the line joining fiducial peaks.	PQ,QR,RS,ST,QS,PT,PS,QT,PR
Angle	Obtained on the basis of the angles between fiducial points.	PQR,QRS,RST,RQS,RSQ,RTS
Miscellaneous	Obtained as areas, ratios, perimeters, in-radius, and centroids.	$QRS_{area},\frac{QRS_{area}}{RS^{2}},{\left(\frac{R}{S}\right)}_{∠},\frac{R_{∠}}{QS_{time}},$ $\frac{S_{∠}}{QT_{time}},\frac{S_{∠}}{PQ},{\left(\frac{R}{Q}\right)}_{∠},{\left(\frac{R}{T}\right)}_{∠},{\left(\frac{Q}{T}\right)}_{∠},$ $\frac{QRS_{area}}{Q_{a}R_{a}},QRS_{perimeter},QRS_{inradius},$ $QRS_{X_{centroid}},QRS_{Y_{centroid}}$

## Abbreviations

The following abbreviations are used in this manuscript:

AC	Autocorrelation
ACC	Accuracy
AP	Action Potential
AC/DCT	Autocorrelation/Discrete Cosine Transform
CNN	Convolutional Neural Network
DNN	Deep Neural Network
DCT	Discrete Cosine Transform
DT	Decision Tree
DL	Deep Learning
ECG	electrocardiogram
EER	Equal Error Rate
GM	Gaussian Mixture
HRV	Heart Rate Variability
ICA	Independent Component Analysis
*k*-NN	*k*-Nearest Neighbor
LDA	Linear Discriminant Analysis
LDAC	Linear Discriminant Analysis Classifier
LLR	Log-Likelihood Ratio
ML	Machine Learning
MLP	Multi-Layer-perceptron
NN	Neural Network
NSR	Normal Sinus Rhythm
PCA	Principal Components Analysis
PNN	Probabilistic Neural Network
QDA	Quadratic Discriminant Analysis
RBFNN	Radial Basis Function Neural Network
RF	Random forest
RNN	Recurrent Neural Network
SIMCA	Soft Independent Modelling of Class Analogy
SVD	Singular Value Decomposition
SVM	Support Vector Machine

## Appendix A. ECG Recognition Studies

**Table A1:** Comprehensive list of works using ECGs for biometric recognition.

Ref ^a^	Year	DB ^b^	NI ^c^	FT ^d^	FR ^e^	CT ^f^	CP ^g^
Gong et al. [125]	2024	MIT-BIH, PTB, and DUNK ^1^	441 ^2^	NF	-	DRCN and pretrained DNNs ^3^	$acc_{max}≃95.77\%$
Al-Jibreen et al. [207]	2024	MIT-BIH	47	NF	-	CNN	$acc_{max}≃99.58\%$
Liu et al. [137]	2024	MIT-BIH, ECG-ID, and PTB	648 ^4^	NF	TVPCAD ^5^	RLRLR ^6^	$acc_{max}≃100\%$
Carvalho and Brás [211]	2024	MIT-BIH, Emote_1, and Emote_2	130	F/NF	PCA	RF ^7^	$acc_{max}≃100\%$
Aslan and Choi [208]	2024	CU-ECG	100	NF ^8^	-	GIN ^9^	$acc_{max}≃99.55\%$
Wu et al. [200]	2023	PTB and CYBHi	348	NF ^10^	-	CNN	$eer_{min}=1.96\%$ $acc_{max}≃99.7\%$
Li et al. [174]	2023	Multiple ^11^	373	NF	-	Multiple ^12^	$acc_{max}≃100\%$
Wang et al. [188]	2023	ECG-ID, MIT-BIH, and USSTDB	373	NF ^13^	-	*k*-NN ^14^	$acc_{max}≃100\%$
Yi et al. [201]	2023	PTB, ECG-ID, CYBHi, and Heartprint	642	NF ^15^	-	ADAFFN ^16^	$acc_{max}≃92.58\%$
Zhang et al. [209]	2023	PTB, ECG-ID, and CYBHi	445/443	NF	-	EfficientNet ^17^	$precision_{max}≃99.6\%$
Zhang et al. [187]	2023	ST-T	20/50/70	NF ^18^	-	*k*-NN ^19^	$acc_{max}≃99.14\%$
Bhuva and Kumar [173]	2023	PTB ^20^	N/A	F/NF	-	Multiple ^21^	$acc_{max}≃100\%$
Aleidan et al. [210]	2023	ECG-ID	90	NF ^22^	-	LightGBM	$acc_{max}≃98.7\%$
Chan et al. [202]	2023	PTB	115	NF ^23^	-	CNN	$acc_{max}≃99.63\%$
Ammour et al. [203]	2023	Heartprint	199	NF ^24^	-	CNN	$acc_{max}≃98.02\%$
Meltzer and Luengo [136]	2023	MIT-BIH and PTB	336 ^25^	NF ^26^	PCA and DCT ^27^	*k*-NN ^28^	$acc_{max}≃100\%$
Prakash et al. [204]	2023	ECG-ID	90 ^29^	NF ^30^	-	NN ^31^	$acc_{max}≃99.85\%$
Biran and Jeremic [186]	2023	PTB	62	F/NF ^32^	-	*k*-NN	$acc_{max}≃97.03\%$
Wang et al. [185]	2023	CYBHi, MIT-BIH, and PTB	358	NF	-	*k*-NN	$eer_{min}=1.06\%$ $acc_{max}≃99.1\%$
D’angelis et al. [205]	2023	CYBHi and Heartprint	262	NF ^33^	-	CNN ^34^	$eer_{min}=0\%$ $acc_{max}≃99.35\%$
Cámara Núnez et al. [206]	2023	Multiple ^35^	43	NF ^36^	-	CNN ^37^	$acc_{max}≃91.6\%$
Melzi et al. [121]	2023	PTB, ECG-ID, CYBHi, and private database	Various ^38^	NF ^39^	-	CNN ^40^	$eer_{min}=0\%$ $acc_{max}≃100\%$
Prakash et al. [118]	2022	Multiple ^41^	1373 ^42^	NF ^43^	-	CNN-LSTM	$acc_{max}=99.62\%$
Prakash [195]	2022	Multiple ^41^	1463	NF: ^44^	-	CapNet ^45^	$acc_{max}=99.40\%$
Hammad et al. [196]	2022	PTB	290	NF	-	CNN	eer=0.47% $acc_{max}=100\%$
Fatimah et al. [172]	2022	MIT-BIH, ECG-ID, and CYBHi	202	NF ^46^	-	Various ^47^	$acc_{max}=98.45\%$
Lee and Kwak [171]	2022	MIT-BIH ^48^, CU-ECG	142	NF ^49^	-	Multiple ^50^	$acc_{max}=98.48\%$
Ibtehaz et al. [158]	2022	Multiple ^51^	508	NF	-	NNs ^52^	$eer_{min}=1.29\%$ $acc_{max}=100\%$
Chee and Ramli [197]	2022	Multiple ^53^	19,270	NF ^54^	-	NN ^55^	$eer_{min}=0.29$ $acc_{max}=100\%$
Kim et al. [122]	2022	ECG-ID	83	NF	-	DNN ^56^	${\bar{acc}}_{max}=99.91\%$
Sun et al. [117]; ^57^	2022	ECG-ID, MIT-BIH, and PTB	- ^58^	NF ^59^	-	*k*-NN ^60^	$acc_{max}=93.3\%$
Wang et al. [183] ^61^	2022	MIT-BIH and CYBHi	110	NF	-	*k*-NN	$eer_{min}=0.02\%$ $acc_{max}=99.7\%$
Li et al. [198]	2022	ECG-ID, and MIT-BIH	156	F	-	WOA-PNN	$acc_{max}=99.43\%$
Jyotishi and Dandapat [199]	2022	PTB, ECG-ID, CYBHi, and UofTDB	1446	NF	-	HLSTM	$eer_{min}=0.89\%$ acc=98.6%
Meltzer and Luengo [184]	2022	MIT-BIH, PTB	336 ^62^	NF ^63^	DCT ^64^	*k*-NN ^65^	$acc_{max}≃100\%$
Hwang et al. [170]	2021	n/a ^194 66^	15	F ^67^	-	Various ^68^	${\bar{eer}}_{min}=0.77\%$ ${\bar{acc}}_{max}=99.05\%$
Zhang et al. [191]	2021	Multiple ^69^	600	NF	-	CNN-TL ^70^	acc=99.17/100%
Tirado-Martin and Sanchez-Reillo [192]	2021	n/a ^194^	104	NF ^71^	-	NN ^72^	$eer_{min}=0\%$ $acc_{max}=98.19\%$
AlDuwaile and Islam [193]	2021	PTB and ECG-ID	190	NF ^73^	-	CNN ^74^	$acc_{max}=99.90\%$
Sepahvand and Abdali-Mohammadi [182]	2021	PTB and CYBHi	20	NF ^75^	-	2 types ^76^	$eer_{min}=1.41\%$ $acc_{max}=99\%$
Zhou et al. [169]	2021	n/a ^194^	23	F/NF ^77^	-	SVM	$acc_{max}=97\%$
Srivastva et al. [194]	2021	PTB and CYBHi	176	NF ^78^	-	PlexNet ^79^	$acc_{max}=99.66\%$
Ingale et al. [181]	2020	Multiple ^80^	1694	F/NF	-	*k*-NN and DTW	$\bar{eer}=2.11\%$ $acc_{bestcase}=100\%$
Hong et al. [139]	2020	CH ^81^	8528	NF ^82^	-	*k*-NN ^83^	$acc_{max}=80.4\%$
Li et al. [127]	2020	FANTASIA, CEBSDB, NSRDB, STDB, and AFDB	129	NF	-	CNN ^91^ and Cascaded CNN	acc=94.3/97.1%
Barros et al. [226]	2020	Physionet Challenge ^84^	1985	F/NF	-	RF ^7^	$F1_{max}=88\%$
Wang et al. [138]	2019	ECG-ID and MIT-BIH	137 ^85^	NF ^86 113^	-	NN ^87^	$acc_{max}=100\%$
Labati et al. [115]	2019	PTB and THEW ^88^	52 ^89^	NF ^90^	-	CNN ^91^	$eer_{min}=1.05\%$ $acc_{max}=100\%$
Aziz et al. [168]	2019	n/a ^194^	14	NF ^92^	-	SVM, *k*-NN, and DT ^93^	$acc_{max}=98.72\%$ (SVM-C)
Meltzer and Luengo [44]	2019	PTB	52	F	Multiple ^94^	Multiple ^95^	acc>99.65%
Dong et al. [113]	2018	PTB	113	NF ^96^	-	*k*-NN ^97^	acc=95.6/100%
Bassiouni et al. [157]	2018	ECG-ID and MIT-BIH	120	F/NF ^183 98^	DCT ^183^	SVM, NN, and *k*-NN	$acc_{max}=99\%$ (ECG-ID), $acc_{max}=100\%$ (MIT-BIH)
Šprager et al. [180]	2017	n/a ^194^	4	NF ^99^	SVD ^100^	*k*-NN	$eer_{min}=5\%$ $acc_{max}=99\%$
Salloum and Kuo [111]	2017	ECG-ID and MIT-BIH	- ^101^	NF ^102^	-	RNN ^103^	eer→0 as the training% increases
da Silva Luz et al. [112]	2017	CYBHi and UofT	- ^104^	NF ^105^	-	CNN ^91^	$eer_{min}=1.33\%$
Zhang et al. [190]	2017	Multiple ^106^	220	NF ^107^	-	NN	acc=93.5%
Zhang et al. [155]	2017	n/a ^194^	10	NF ^108^	-	NN	98.4%
Pinto et al. [156]	2017	n/a ^194^	- ^104^	NF ^109^	-	SVM, *k*-NN, MLP, GM ^110^	$eer_{min}=2.66\%$, $acc_{max}=94.89\%$
Ciocoiu [179]	2017	PTB and CYHBi	100	NF ^111^	-	*k*-NN	$acc_{max}=100\%$
Tan and Perkowski [178]	2017	MIT-BIH ^112^, ECG-ID	184	F/NF ^113^	-	RF and *k*-NN ^114^	up to 99.52%
Louis et al. [221]	2016	UofT	1012	NF ^115^	-	BA ^116^	$eer_{min}=7.89\%$
Hejazi et al. [167]	2016	n/a ^194^	52	NF ^117^	Multiple ^118^	SVM	$eer_{min}=2.46\%$
Lei et al. [166]	2016	PTB	200	NF	-	SVM and CNN	$acc_{max}=99.33\%$
Gutta and Cheng [165]	2015	MIT-BIH	18	NF ^183^	DCT ^183^	GLM ^119^; SVM	$acc_{max}<95\%$
Sarkar et al. [153]	2015	MIT-BIH	47	NF	-	LDAC, *k*-NN, and QDC ^120^	up to 97%
Singh [163]	2015	QT, Priv ^121^	191	F ^122^	FLD ^123^	*k*-NN	$err_{min}=0.71\%$
Arteaga-Falconi et al. [242]	2015	Multiple ^124^	73	F	-	Priv ^125^	$TAR_{max}=84.93\%$, $FAR_{min}=1.29\%$
Lin et al. [152]	2014	n/a ^194^	26	NF ^126^	-	SVM	$acc_{max}=81.73\%$
Carreiras et al. [177]	2014	n/a ^194^	618	F	-	*k*-NN ^127^	eer=9.01%
Da Silva et al. [164]	2013	n/a ^194^	63	NF ^128^	-	SVM and *k*-NN	$eer_{min}=0.99\%$
Gürkan et al. [176]	2013	PTB	30	NF ^129^	DCT ^130^	*k*-NN ^131^	$acc_{max}=97.31\%$
Tawfik and Kamal [48]	2011	n/a ^194^	22	NF ^132^	DCT	NN ^186^	99.09%
Safie et al. [104]	2011	PTB	112 ^133^	NF ^134^	-	*k*-NN	$eer_{healthy}=18.12\%$, $eer_{arrhyth}=26.58\%$
Lourenço et al. [151]	2011	n/a ^194^	16	NF ^134^	Obs ^194 135^	*k*-NN	$eer_{min}=13\%$ acc = 94.3%
Shen et al. [32]	2011	n/a ^194^	168	F	-	*k*-NN ^136^	eer<0.05
Ye et al. [107]	2010	MIT-BIH ^137^, ST ^137^	Multiple ^138^	NF ^139^	PCA	SVM	up to 99.6%
Venkatesh and Jayaraman [150]	2010	MIT-BIH	15	F	-	DTW, FLDA ^140^	$acc_{max}$ = 100%
Odinaka et al. [108]	2010	PTB and MIT-BIH	269	NF ^141^	-	Cust ^142^	$eer_{min}=0.37\%$, $acc_{max}$ = 100%
Loong et al. [99]	2010	n/a ^194^	15	NF ^143^	-	NN ^186^	$acc_{max}$ = 100%
Li and Narayanan [102]	2010	MIT-BIH	18	NF ^144^	HLDA ^145^	SVM and GMM ^146^	$eer_{min}$ = 0.5% acc = 98.3%
Jang et al. [148]	2010	n/a ^194^	65	NF ^147^	-	LDAC ^193^	acc→100%
Ghofrani and Bostani [105]	2010	PTB	12	NF ^148^	-	Multiple ^149^	$acc_{max}=100\%$
Coutinho et al. [40]	2010	n/a ^194^	19	NF ^150^	-	*k*-NN ^151^	$acc_{max}=100\%$
Agrafioti and Hatzinakos [85]	2010	n/a ^152^	52	NF ^153^	-	*k*-NN	≤92.3%
Sufi et al. [149]	2010	n/a ^194^	15	NF ^154^	-	*k*-NN ^155^	$acc_{max}=100\%$
Irvine and Israel [243]	2009	n/a ^194^	104	F	-	Cust ^156^	93.1%
Fang and Chan [89]	2009	n/a ^194^	100	NF ^157^	-	*k*-NN ^158^	$acc_{max}=99\%$
Boumbarov et al. [147]	2009	n/a ^194^	9	NF ^159^	- ^159^	NN ^160^	≤94%
Fatemian and Hatzinakos [39]	2009	PTB, MIT-BIH	27	NF ^161^	TR ^162^	*k*-NN ^163^	99.63%
Yao and Wan [146]	2008	n/a ^194^	20	NF ^164^	-	Cust	-
Wan et al. [145]	2008	n/a ^194^	23	NF ^165^	-	NN ^186^	100%
Singh and Gupta [45]	2008	QT ^171^ and MIT-BIH ^166^	35	F	-	*k*-NN	$acc_{max}\;=\;99\%$
Khalil and Sufi [144] ^167^	2008	n/a ^194^	10	NF ^168^	-	-	-
Irvine et al. [129]	2008	n/a ^194^	39	NF	PCA	*k*-NN ^169^	100%
Gahi et al. [162]	2008	n/a: ^194^	16	F	IGR ^170^	LDAC ^193^	100%
Chiu et al. [143]	2008	QT ^171^, n/a	35+ 10 ^172^	NF ^173^	-	*k*-NN	95.71% (81%) FRR = 0.86% (5.11%)
Agrafioti and Hatzinakos [82]	2008	PTB and MIT-BIH	27	NF ^183 174^	DCT ^183^; LDA ^174^	*k*-NN	eer < 1% 100% (AC/LDA), 96.3% (AC/DCT)
Agrafioti and Hatzinakos [83]	2008	PTB	14	NF ^174^	LDA ^174^	Cust ^175^	100%
Chan et al. [38]	2008	n/a ^194^	50	F	SAECG ^176^	*k*-NN ^177^	$acc_{max}=95\%$
Wang et al. [81]	2007	PTB and MIT-BIH	26	F/NF ^183^	DCT ^183^; PCA	Cust ^178^	up to 100%
Wübbeler et al. [142]	2007	PTB	74	NF	-	*k*-NN	$eer_{min}=3\%$ 99.0%
Silva et al. [175]	2007	n/a ^194^	26	F	FS ^179^	Cust ^180^	98.09–99.07%
Molina et al. [141]	2007	n/a ^194^	10	NF	DTW ^181^	Cust	$eer_{min}=2\%$
Zhang and Wei [161]	2006	n/a ^194^	502	F	PCA	Cust ^182^	$acc_{max}=97.4\%$
Plataniotis et al. [78]	2006	PTB	14	NF ^183^	DCT ^183^	Cust ^184^	$FN_{max}=0.0106$ NED $FN_{max}=5.2\times 10^{-4}$ NGLL
Kim et al. [47]	2006	n/a ^194^	10	F	-	LDAC ^193^	-
Saechia et al. [93]	2005	n/a ^194^	-	F	FC ^185^	NN ^186^	$FR_{max}=20\%$
Israel et al. [31]	2005	n/a ^194^	29	F	SWFS ^192^	LDAC ^193^	98–100%
Palaniappan and Krishnan [30]	2004	MIT-BIH	10	F	-	NN ^186 187^	$\bar{acc}=96.17\%$
Kyoso [35]	2003	n/a: ^194^	21	F ^188^	-	LDAC ^193^	corr. > 90% ^189^
Irvine et al. [160]	2003	MIT-BIH	36	F	SWFS ^192^	LDAC ^193^	88–91%
Shen et al. [189]	2002	MIT-BIH	20	F	-	NN ^190^	$id_{rate}$: 80–100%
Kyoso and Uchiyama [28]	2001	n/a ^194^	9	F	-	LDAC ^193^	acc>90% ^191^
Irvine et al. [159]	2001	n/a ^194^	36	F	SWFS ^192^	LDAC ^193^	-
Biel et al. [3]	2001	n/a ^194^	30	F	PCA	SIMCA ^195^	45–50/50

^1^ DUNK dataset: students at an unidentified North Korean university. ^2^ 47 subjects in the MIT-BIH database, 294 subjects in the PTB database, and 100 from the DUNK database. ^3^ CNN, ResNet, AlexNet, MobileNet, and VGG16. ^4^ All subjects in the MIT-BIH, ECG-ID, and PTB databases. ^5^ PCA with total variation regularization (TVPCAD). ^6^ Regularized label relaxation linear regression method (RLRLR) [244]. ^7^ Random forest Classifier. ^8^ Graph description. ^9^ Graph Isomorphism Neural Network. ^10^ Feature vectors obtained from heartbeats using a CNN. ^11^ DREAMER, DECAF, HCILAB, and ECG-ID. ^12^ SVM, Deep NN, and *k*-NN with different metrics. ^13^ Convolutional Network as feature extractor. ^14^ Cosine similarity metric. ^15^ Multibranch NN. ^16^ Attention-Enhanced Domain Adaptive Feature Fusion Network. ^17^ Multiple CNN models followed by voting scheme. ^18^ Max pooling of sparse feature coefficients obtained from dictionary obtained by using KSVD algorithm. ^19^ With k=1 and an unmentioned distance metric. ^20^ this study uses also an electromyography database for multibiometric operation. ^21^ CNN, Decision tree, and SVM. ^22^ First, stage feature vectors are obtained from 2D spectrogram images of the ECGs by means of transfer learning over the pretrained VGG-16 network. Second, stage features are obtained from the first-stage ones by using an RNN-LSTM model. ^23^ Phase space reconstruction as features. ^24^ Features are extracted from 2D time/frequency matrices of heartbeats; extraction is performed in two stages: first, a custom convolutional network, and then, an EfficientNet pretrained model. ^25^ All subjects in the MIT-BIH database and all subjects in the PTB database except one. ^26^ Single-heartbeat samples as features and AC coefficients of 5 s non-overlapped slots. ^27^ For reducing heartbeat sample features and AC sequences of heartbeats samples; also, reduction in examples by using prototypes. ^28^ Multiple metrics. ^29^ Lead I only. ^30^ 2D images of ECG beats. ^31^ Siamese CNNs, Euclidean comparison of vectorized features outputs and sigmoid units output layer. ^32^ 6 fiducial features and Short-Time Fourier Transform (STFT) coefficients. ^33^ 2D images of averaged heartbeats. ^34^ Classifier based on the Vision Transformer (ViT) first presented by Dosovitskiy et al. [245]. ^35^ MIT-BIH Normal Sinus Rhythm Database and Glasgow University High Precision ECG Database (ECG- GUDB). ^36^ 2D representation using Gramian Angular Field (GAF) [246]. ^37^ Custom convolutional network inspired by the VGG19 network. ^38^ Three different experiments using the private database for training and the rest of the databases for testing. ^39^ Variational autoencoder for feature extraction. ^40^ Different architectures for identification and identity verification. ^41^ ECG-ID, PTB, CYBHi, and UofTDB. ^42^ Not explicitly described in the work. ^43^ Discrete wavelet transform (DWT) as described in Kabir and Shahnaz [247]. ^44^ heartbeats converted into gray-scale images. ^45^ CNN with capsule and label layers as described in Jiao et al. [248]. ^46^ Autoregressive coefficients of order 5 of the phase transform, energy distribution of the $i^{th}$ Fourier intrinsic band functions, skewness, and kurtosis of ECG frames. ^47^ Random Forest, ensemble subspace discriminant, and SVM with linear, quadratic, and cubic kernels. ^48^ Not mentioned which one, but it seems to be the Arrhythmia Database given that the paper states that it contains records from 47 subjects. ^49^ PCANet, ICANet1, and ICANet2. ^50^ Decision Tree, *k*-NN, SVM, and two (not described) NNs. ^51^ ECG-ID, MIT-BIH Arrhythmia, PTB, MIT-BIH NSRDB, and CYBHi. ^52^ CNN and Siamese NN for identification and identity verification, respectively. ^53^ Apnea-ECG database, Long-Term AF Database, MIT-BIH Arrhythmia Database, MIT-BIH Long-Term ECG Database, MIT-BIH Malignant Ventricular Ectopy Database, MIT-BIH Polysomnographic Database, MIT-BIH Supraventricular Arrhythmia Database, St Petersburg INCART 12-lead Arrhythmia Database, Fantasia Database, and PTB-XL. ^54^ Features are extracted from normalized segments by using a 1D CNN. ^55^ Fully connected layers for both identification and identity verification. ^56^ This paper presents a framework and tests it with two different NNs: an RNN and a CNN. ^57^ This study focuses on continuous identification only. ^58^ Not mentioned; it is assumed that all subjects available in each database are used. ^59^ Trained autoencoder as subject generative feature. ^60^ Using reconstruction error as metric. ^61^ This is the only known work to date to use an Online ML model updating technique. ^62^ All subjects in the MIT-BIH database and all subjects in the PTB database except one. ^63^ Single-heartbeat samples as features and AC coefficients of 5 s non-overlapped slots. ^64^ For reducing heartbeat sample features and AC sequences of heartbeat samples; observation reduction was also applied. ^65^ Multiple metrics. ^66^ ECG-ID for heart rate analysis. ^67^ 29 fiducial points of nonlinear normalization of consecutive $P_{on}$ bounded segments according to heart rate. ^68^ SVM, *k*-NN, and Decision Tree. ^69^ AF Classification from a Short Single Lead ECG Recording Database, Improving the Quality of ECGs Collected using Mobile Phones Database, ECG-ID, and CYBHi; performance comparisons with the PTB and CYBHi databases. ^70^ Deep transfer learning and a convolutional neural network. ^71^ 2D matrix of a fixed number of hear beats, each with a fixed number of ECG samples. ^72^ CNN with an LSTM layer ^73^ 2D matrix formed by using continuous wavelet transformation of ECG segments with different time scales. ^74^ Other pretrained DNs used for comparison purposes. ^75^ Output of multi-lead ECG segments preprocessed and passed to multi-CNN-genes. ^76^ Fully connected NN for identification and a simple *k*-NN classifier using the Euclidean metric for identity verification. ^77^ Engineered features both fiducial and non-fiducial, and learned features using a CNN; both conform a feature vector of an ECG segment. ^78^ 2D image of ECG segments. ^79^ Ensemble of preexisting fine-tuned architectures. ^80^ PTB, MIT-BIH, CEBSDB, CYBHi, ECG-ID, and a private database (ECG-BG). ^81^ AF Classification from a Short Single Lead ECG Recording Database. ^82^ Deep autoencoder results as features. ^83^ Using hamming distance, k = 1, and a decision threshold. ^84^ Improving the Quality of ECGs Collected using Mobile Phones challenge database. ^85^ 90 from ECG-ID and 47 from MIT-BIH. ^86^ Feature learning using a sparse autoencoder stack. ^87^ Autoencoder-based NN classifier. ^88^ Telemetric and Holter ECG Warehouse of the University of Rochester (THEW). ^89^ 276 simulated subjects. ^90^ Outputs of a CNN as features given heartbeats as inputs. ^91^ Convolutional Neural Network. ^92^ Shannon energy, skewness, variance, occupied bandwidth, and median frequency. ^93^ Decision Tree. ^94^ Step-wise based on Wilks’ lambda and PCA. ^95^ SVM, *k*-NN, LDA, and QDA. ^96^ ECG system dynamics as features. ^97^ L1 norm of bank of errors $V^{k}$. ^98^ discrete wavelet transform of mean heartbeat. ^99^ higher-order statistics of each heartbeat as features [249]. ^100^ Singular Value Decomposition (SVD) [249]. ^101^ Not mentioned; it can be interpreted that all subjects for both databases are used. ^102^ concatenated normalized samples of a number of heartbeats as features. ^103^ Recurrent Neural Network (RNN). ^104^ Not mentioned. ^105^ outputs of a CNN as features given ECGs and spectrograms as inputs. ^106^ combined measurement of ECG, Breathing and Seismocardiograms, WECG [250], Fantasia, MIT-BIH Normal Sinus Rhythm, MIT-BIH ST Change, MIT-BIH, MIT-BIH Atrial Fibrillation, and MIT-BIH Malignant Ventricular Ectopy. ^107^ Multistage feature extraction using windowing, wavelet transform, autocorrelation, component selection, and CNN for feature learning. ^108^ Outputs of a CNN as features given 2D transformations of the ECG as inputs. ^109^ Discrete Cosine Transform and Haar Wavelet transform. ^110^ MLP, GM. ^111^ Discrete Cosine Transform, discrete wavelet transform, random projections, and encoding using a codebook as features. ^112^ Arrhythmia and normal sinus rhythm. ^113^ Discrete wavelet transform. ^114^ Random Forest applied to fiducial features and *k*-NN to non-fiducial features. ^115^ One-Dimensional Multi-Resolution Local Binary Patterns as features. ^116^ Bootstrap Aggregating: Breiman [251]; GM. ^117^ Autocorrelation. ^118^ PCA, LDA, and KPCA. ^119^ Generalized Linear Model (see McCullagh [252]). ^120^ Quadratic Discriminant Classifier (QDA). ^121^ Private database of the Indian Institute of Technology, Banaras Hindu University, Varanasi, India. ^122^ Heartbeat interval, interbeat interval, and heartbeat morphological features. ^123^ Fisher’s Linear Discriminant Analysis. ^124^ ST-T, MIT-BIH, QT, and privately recorded records. ^125^ Hierarchical validation scheme; sequential algorithm based on the Nearest Neighbor technique. ^126^ Root mean square value, nonlinear Lyapunov exponent, and correlation dimension. ^127^ With cosine distance metric and k = 3. ^128^ Mean or median signal of 5 heartbeats as feature/template. ^129^ Autocorrelation, Mel-Frequency Cepstrum Coefficients, and vector of samples R centered. ^130^ Only for reducing the AC sequences. ^131^ Euclidean metric and k=3. ^132^ Time- and amplitude-normalized heartbeats as features. ^133^ 98 diagnosed with arrhythmia, the rest without pathologies. ^134^ Mean time- and amplitude-normalized heartbeats per subject as features. ^135^ Each subject is represented by a single normalized mean heartbeat. ^136^ Minimum Euclidean distance-based classifier. ^137^ Arrhythmias, Normal Sinus Rhythm, and Long-term ST databases. ^138^ Long/short term Normal Sinus Rhythm Database: 18; Arrhythmias: 47; combined: 65. ^139^ Concatenated Daubechies wavelet coefficients and Independent Component Analysis (ICA) components [253]. ^140^ Dynamic Time Warping, and Fisher’s Linear Discriminant Analysis with Nearest Neighbor Classifier. ^141^ Short-time Fourier transform components of normalized ECG heart pulse as features. ^142^ Generative classifier. ^143^ Linear predictive coding coefficients and wavelet packet decomposition coefficients as features. ^144^ Cepstral and Hermite polynomial expansion features. ^145^ Heteroscedastic LDA, only for cepstral features [254]. ^146^ With score fusion. ^147^ Principal components coefficients as features. ^148^ Autoregressive coefficients, Higuchi dimension, Lyapunov exponent, and Shannon approximation entropy as different features. ^149^ *k*-NN, MLP, and Probabilistic Neural Network (PNN). ^150^ Incremental LZ parsing algorithm outputs as features. ^151^ Incremental LZ cross-parsing algorithm output as distance metric (minimum incremental coding length). ^152^ Not mentioned, but previous works by the authors use the PTB database and the number of subjects in this work coincides with the number of subjects of the control group in that database. ^153^ AC/LDA and Periodicity Transform. ^154^ Polynomial coefficients as features. ^155^ L1-based norm distance comparison as classifier. ^156^ Sequential test statistic classifier. ^157^ Phase space reconstruction as features. ^158^ Spatial correlation and mutual nearest-point distance as distance metrics. ^159^ PCA principal components and LDA coefficients as features; no reduction applied. ^160^ Radial Basis Function Neural Network (RBFNN). ^161^ Discrete wavelet transform. ^162^ Template reduction using the median of normalized heartbeats. ^163^ With template correlation similarity comparison. ^164^ Wavelet coefficients matrix as features. ^165^ Interpolated samples of each RR interval. ^166^ 15 from MIT-BIH Arrhythmia database and 10 from MIT-BIH Normal Sinus database. ^167^ Demonstration of ECG features as keys, no classification performed. ^168^ Legendre polynomials coefficients. ^169^ With features distance as dissimilarity metric. ^170^ Feature selection on the basis of the Information Gain Ratio [255]. ^171^ QT database. ^172^ 10 individuals diagnosed of arrhythmia from the QT database. ^173^ Discrete wavelet transform coefficients. ^174^ Autocorrelation coefficients as features and dimensionality reduction as LDA coefficients of features. ^175^ Decision fusion of *k*-NN classifiers. ^176^ signal-averaged ECG. ^177^ PRD, CCORR and WDIST based distances classifiers. ^178^ Hierarchical classifier schema with LDAC and NN stages. ^179^ Wrapper sequential forward search feature selection [256,257]. ^180^ Multiple *k*-NN classifiers and majority voting stage. ^181^ Dynamic Time Warping [258]. ^182^ Bayes’ probabilistic-based and discriminant analysis classifiers. ^183^ Autocorrelation coefficients as features and dimensionality reduction as Discrete Cosine Transform coefficients of features. ^184^ Normalized Euclidean and Normalized Gaussian Log Likelihood distance-based classifiers. ^185^ Fourier Coefficients of time domain P, QRS and T complexes. ^186^ Neural Network. ^187^ MLP-BP [259], and SFA (Vakil-Baghmisheh and Pavešić [260]). ^188^ See Kyoso and Uchiyama [261]. ^189^ Registered/unregistered. ^190^ Neural Network; see Simon and Eswaran [262]. ^191^ In some experiments. ^192^ Step-wise features selection. ^193^ Linear Discriminant Analysis Classifier (LDAC). ^194^ Private database. ^195^ See Esbensen et al. [263]. ^a^ Reference to the published work. ^b^ Used databases (see Table A2). ^c^ Number of individuals involved. ^d^ Type of features: fiducial (F) or/and non-fiducial (NF). ^e^ Feature reduction type (when defined). ^f^ Classifier type. ^g^ Classifier performance.

## Appendix B. ECG Databases

**Table A2:** Comprehensive list of the databases containing ECGs that have been referred to as used in published research works.

Database	Pub.	Sampling Freqs.	Available Leads	Length of Recordings	Number of Subjects
Emote_1&2 [211]	2024	1 KHz	N/A ^196^	N/A	30 and 53
DUNK [125]	2024	N/A	1	10 s	100
La Princesa University Hospital (Madrid, Spain) [121]	2023	500 Hz	Various ^197^	N/A	81,974
USSTDB [188]	2023	500 Hz	1	20 s	60
Heartprint [241]	2022	250 Hz	1 ^198^	15 s	199
PTB-XL, a large, publicly available electrocardiography dataset [16,264]	2022	1 KHz	12	10 s	18,869 (21,799 records)
VitalDB ^199^ [265]	2022	500 Hz	Varying	90 min to 240 min	6388
Chosun University CU-ECG [171,208]	2022	500 Hz ^200^	1	10 s	95 ^200^
CapnoBase IEEE TBME Respiratory Rate Dataset ^201^ [266]	2021	300 Hz	1	8 min	42
Lobachevsky University Electrocardiography Database (LUDB) [16,267,268]	2021	500 Hz	12	10 s	200
Psychological stress states [169]	2021	250 Hz	N/A ^202^	3.5 s ^203^	23
Fetal electrocardiograms – B1 Pregnancy Dataset [269]	2020	500 Hz	4 abdominal	20 min	10
Fetal electrocardiograms – B2 Labour Dataset [269]	2020	500 Hz–1 KHz	4 abdominal + 1 direct fetal	5 min	12
Chapman University and Shaoxing People’s Hospital Database [270]	2020	500 Hz	12	10 s	10,646
MARSH Database [271]	2020	N/A	2	15 min	29
Atrial Fibrillation Screening Database [272]	2020	300 Hz	1	40 s	2422
Non-Invasive Fetal ECG Arrhythmia Database [16,273]	2019	500 Hz–1 KHz	4–5 abdominal + 1 chest maternal	7 min to 32 min	26 (14 healthy and 12 with arrhythmia)
Continuously Annotated Signals of Emotion (CASE) [274]	2019	1 KHz	2 or 3	40 min	30
Cognitive Load, Affect and Stress (CLAS) [275]	2019	256 Hz	1	30 min	62
The China Physiological Signal Challenge 2018 [276]	2018	500 Hz	12	6 s to 60 s	6877
ECG-Fitness Database [277]	2018	n/a ^204^	2	1 to 2 min	17
Glasgow University High Precision ECG Database [278]	2018	250 Hz	3	10 min	25
Wearable stress and affect detection (WESAD) [279]	2018	700 Hz	2	≈36 min	15
AMIGOS Dataset [280]	2018	256 Hz	N/A	N/A	40
BIDMC PPG and Respiration Dataset ^205^ [16,281]	2018	125 Hz	1 ^206^	8 min	53
Wrist PPG During Exercise [16,282]	2017	256 Hz	1	Up to 10 min	8
AF Classification from a Short Single Lead ECG Recording Database ^207^ [16,283]	2017	300 Hz	1	9 s to 60 s	8528
Preterm Infant Cardio-Respiratory Signals Database ^208^ [16,284]	2017	250 Hz and 500 Hz	1	20 h to 70 h	10
iAMwell ^209^ [285]	2017	2 KHz	2 or 3	N/A	15
DREAMER [286]	2017	256 Hz	1	Varying	25
MIMIC-III Clinical Database ^208^ [16,287]	2016	125 Hz	Different leads for each record	Varying, up to several weeks	53,423
Motion Artifact Contaminated ECG Database [16,288]	2015	500 Hz	4	20 s	1
Vortal Dataset [289]	2016	500 Hz	1 ^206^	10 min	57
DECAF [290]	2015	1 KHz	2	60 min	30
IIT(BHU) Database ^210^	2015	1 KHz	1 ^206^	3–5 min	106
CEBSDB ^211^ [16,291]	2014	5 KHz	2 ^212^	5 and 50 min	20
ECG-ID Database [16,240]	2014	500 Hz	1	20 s	90 (310 records)
University of Toronto ECG Database (UofTDB) [239]	2014	200 Hz	1	2-5 min	1020
Combined measurement of ECG, Breathing, and Seismocardiograms [16,291]	2014	5 KHz	2	1 h to 10 h	20
HCILAB [292]	2013	1 KHz	1 ^213^	≈ 30 min	10
Noninvasive Fetal ECG Database ^214^ [16]	2013	1 KHz	1 abdominal	1 min	75 training + 100 testing
CYBHi [238]	2013	1 KHz	2	5 min/2 sessions	63
Abdominal and Direct Fetal ECG Database [16,293]	2012	1 KHz	4 abdominal + 1 direct fetal	10 min	5
Improving the Quality of ECGs Collected using Mobile Phones ^215^ [16]	2011	500 Hz	12	>10 s	2000
University of Toronto Combined ECG and PCG [294]	2010	200 Hz	1 ^216^	3 min	21
European ST-T Database [16,295]	2009	250 Hz	2	2 h	79 (90 records)
Telemetric and Holter ECG Warehouse (THEW) Databases ^217^ [296]	2008	180 Hz to 1 KHz	3 or 12	Varying	More than 3700
Long-Term AF Database [16,297]	2008	128 Hz	2	24 h	84
St Petersburg INCART 12-lead Arrhythmia Database [16]	2008	257 Hz	12	30 min	75
MIMIC II Database [16,298,299]	2008	125 Hz	12	1 h to 10 h	32,536
Stress Recognition in Automobile Drivers [16,300]	2008	496 Hz	1	50 min to 90 min	17
CU Ventricular Tachyarrhythmia Database [16,301]	2007	250 Hz	1	8 min	35
MIT-BIH Arrhythmia Database [16,229]	2005	360 Hz	2 (MLII and V1/2/4/5)	30 min	47 (48 records)
Sudden Cardiac Death Holter Database [16,235]	2004	250 Hz	2	Up to 24 h	23
PTB Diagnostic ECG Database [16,33]	2004	1 KHz	15	from 10 s to 2 min	290 (549 records)
Intracardiac Atrial Fibrillation Database [16]	2003	1 KHz	5 endocardial + 3 surface	1 min	8
Fantasia Database [16,302]	2003	250 Hz	1	2 h	40
MGH/MF Waveform Database [16,303]	2002	360 Hz	3	12–86 min	250
Apnea-ECG database [16,304]	2000	100 Hz	1	7–10 h	32
MIT-BIH Atrial Fibrillation Database [16,230]	2000	250 Hz	2	10 h	25
Long-Term ST Database [16,305]	2000	250 Hz	2 or 3	21 to 24 h	80 (86 records)
BIDMC Congestive Heart Failure Database [16,306]	2000	250 Hz	2	20 h	15
MIT-BIH Normal Sinus Rhythm Database [16]	1999	360 Hz	2	Up to 24 h	18
MIT-BIH Long Term [16]	1999	360 Hz	2 or 3	14 to 20 h	7
MIT-BIH Supraventricular Arrhythmia Database [16,231]	1999	360 Hz	2 (MLII and V1/2/4/5)	30 min	78
MIT-BIH ST Change Database [16,232]	1999	360 Hz	2	30 min	28
MIT-BIH Noise Stress Test Database [16,233]	1999	360 Hz	2	30 min	12 ECG + 3 noise
MIT-BIH Long-Term ECG Database [16]	1999	128 Hz	2	14-22 h	7
QT Database [16,236]	1999	250 Hz	2	15 min	100
MIT-BIH Polysomnographic Database [16,234]	1999	250 Hz	1	80 h total	16 (18 records)
MIMIC Database ^208^ [16,307]	1996	125 Hz or 500 Hz	Different leads for each record	20, 40 or more hours	90 (121 records)
MIT-BIH Malignant Ventricular Ectopy Database [16,235]	1986	250 Hz	1	30 min	22 records
American Heart Association ECG Database [308]	1980	250 Hz	2	3 h	154

^196^ The BiosignalPlux Explorer Research Kit, which can use up to 4 inputs, is mentioned. ^197^ One or more leads. ^198^ Finger lead. ^199^ Non-cardiac surgery patients during surgery. ^200^ The work [208] refers a sampling rate of 500 KHz, which is very likely to be a mistake, and the same work mentions 100 users, while the previous work [171] mentions 95 subjects. ^201^ First described in 2013 and partially published in 2021. Only ECG R peak positions available. ^202^ Not mentioned in the work. A picture of the equipment suggests a single lead (two electrodes). ^203^ Multiple segments recorded in a period of more than 20 h. ^204^ Taken from a Viatom CheckMe Pro. ^205^ Beth Israel Deaconess Medical Centre (Boston, MA, USA). Signals and numerics extracted from the much larger MIMIC-II matched waveform Database. ^206^ Lead II. ^207^ The PhysioNet/Computing in Cardiology Challenge 2017. ^208^ Recorded from intensive care unit patients. ^209^ Intelligent Athlete Monitoring for Cardiovascular Wellness (iAMwell). ^210^ School of Biomedical Engineering, Indian Institute of Technology (IIT). Banaras Hindu University (BHU), Varanasi, India. ^211^ Combined measurement of ECG, Breathing and Seismocardiograms Database. ^212^ Leads I and II. ^213^ One lead attached to the subject chest. ^214^ The PhysioNet/Computing in Cardiology Challenge 2013. ^215^ The PhysioNet/Computing in Cardiology Challenge 2011. ^216^ Lead I. ^217^ University of Rochester Medical Center.
